# {Bis[5-methyl-3-(trifluoro­meth­yl)pyrazol-1-yl]borato}{tris­[5-methyl-3-(trifluoro­meth­yl)pyrazol-1-yl]borato}cobalt(II): a structure containing a B—H⋯Co agostic inter­action

**DOI:** 10.1107/S1600536810011773

**Published:** 2010-04-10

**Authors:** Robert T. Stibrany, Joseph A. Potenza

**Affiliations:** a Department of Chemistry and Chemical Biology, Rutgers, The State University of New Jersey, 610 Taylor Road, Piscataway, New Jersey 08854 USA

## Abstract

The title compound, [Co(C_10_H_10_BF_6_N_4_)(C_15_H_13_BF_9_N_6_)], is a neutral Co^II^ complex which contains one each of the anionic ligands, bis­(3-trifluoro­methyl-5-methyl­pyrazol-1-yl)borate (Bp) and tris­(3-trifluoro­methyl-5-methyl­pyrazol-1-yl)borate (Tp). A distorted octa­hedral coordination geometry results from ligation of an H atom, which is part of an agostic B—H⋯Co inter­action (H⋯Co = 2.17 Å), and by five imine N atoms, two from a Bp ligand and three from a Tp ligand. In the crystal, mol­ecules form layers parallel to the (10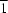) planes, and the layers are linked along the *a* axis by C—H⋯F hydrogen bonds. An intra­molecular C—H⋯F inter­action also occurs.

## Related literature

For our study of nitro­gen-containing heterocyles, such as expanded-ring imidazoles, and their complexes with metal ions, see: Stibrany & Potenza (2009*a* ▶). For metal complexes with pyrazole, see: Stibrany & Potenza (2006 ▶, 2009*b* ▶); Stibrany *et al.* (1999 ▶, 2005 ▶, 2006 ▶). Copper and cobalt complexes utlizing the title ligand have been prepared for oxidation studies, see: Gorun *et al.* (2000 ▶). For agostic inter­actions, see: Ruman *et al.* (2001 ▶, 2002 ▶); Siemer *et al.* (2001 ▶); Ghosh *et al.* (1998 ▶).
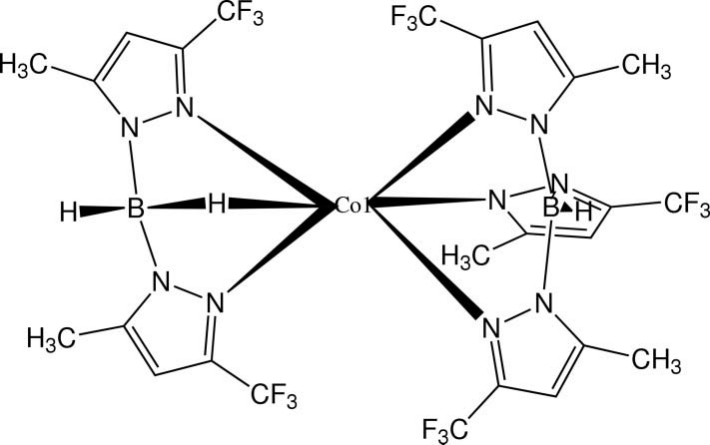


## Experimental

### 

#### Crystal data

[Co(C_10_H_10_BF_6_N_4_)(C_15_H_13_BF_9_N_6_)]
*M* *_r_* = 829.08
Monoclinic, 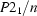

*a* = 10.8195 (16) Å
*b* = 16.559 (2) Å
*c* = 18.687 (3) Å
β = 98.408 (3)°
*V* = 3312.0 (8) Å^3^
*Z* = 4
Mo *K*α radiation
μ = 0.64 mm^−1^
*T* = 100 K
0.37 × 0.30 × 0.14 mm

#### Data collection

Bruker SMART CCD area-detector diffractometer
Absorption correction: multi-scan (*SADABS*; Blessing, 1995 ▶) *T*_min_ = 0.919, *T*_max_ = 1.00
33535 measured reflections
7286 independent reflections
6292 reflections with *I* > 2σ(*I*)
*R*_int_ = 0.024

#### Refinement

*R*[*F* ^2^ > 2σ(*F* ^2^)] = 0.039
*wR*(*F* ^2^) = 0.108
*S* = 1.00
7286 reflections
495 parameters
H atoms treated by a mixture of independent and constrained refinement
Δρ_max_ = 0.69 e Å^−3^
Δρ_min_ = −0.44 e Å^−3^

### 

Data collection: *SMART* (Bruker, 2000 ▶); cell refinement: *SAINT-Plus* (Bruker, 2000 ▶); data reduction: *SAINT-Plus*; program(s) used to solve structure: *SHELXS97* (Sheldrick, 2008 ▶); program(s) used to refine structure: *SHELXL97* (Sheldrick, 2008 ▶); molecular graphics: *ORTEPIII* (Burnett & Johnson, 1996 ▶) and *ORTEP-32* (Farrugia, 1997 ▶); software used to prepare material for publication: *SHELXTL* (Sheldrick, 2008 ▶) and *PLATON* (Spek, 2009 ▶).

## Supplementary Material

Crystal structure: contains datablocks I, global. DOI: 10.1107/S1600536810011773/fk2015sup1.cif

Structure factors: contains datablocks I. DOI: 10.1107/S1600536810011773/fk2015Isup2.hkl

Additional supplementary materials:  crystallographic information; 3D view; checkCIF report

## Figures and Tables

**Table:** 

Co1—N3	2.0850 (17)
Co1—N7	2.1072 (17)
Co1—N9	2.1115 (18)
Co1—N5	2.1351 (17)
Co1—N1	2.1581 (16)
Co1—H22*B*	2.17 (2)
B1—H1*B*	1.10 (2)
B2—H21*B*	1.00 (3)
B2—H22*B*	1.18 (3)

**Table:** 

H21*B*—B2—H22*B*	109.6 (19)
B2—H22*B*—Co1	107 (1)

**Table 2:** Hydrogen-bond geometry (Å, °)

*D*—H⋯*A*	*D*—H	H⋯*A*	*D*⋯*A*	*D*—H⋯*A*
C5—H5*B*⋯F9^i^	0.98	2.48	3.391 (3)	155
C10—H10*C*⋯F11	0.98	2.40	3.160 (3)	134
C25—H25*B*⋯F10^ii^	0.98	2.50	3.414 (4)	155

Symmetry codes: (i) 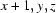; (ii) 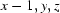.

**Fig. 1.**
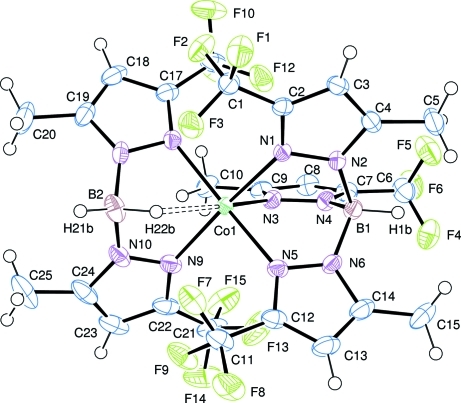
The molecular structure of the title compound showing the atom-numbering scheme. Displacement ellipsoids are shown at the 50% probability level. H atoms are shown as spheres of arbitrary radius.

**Fig. 2.**
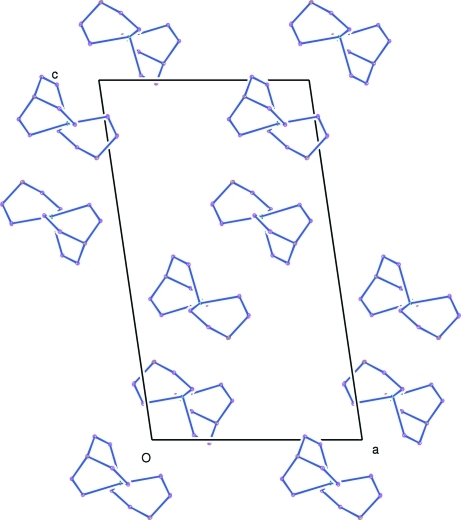
Projection along the b cell direction of the central core of the molecules of (I), showing the layers parallel to the (1 0 -1) planes.

**Fig. 3.**
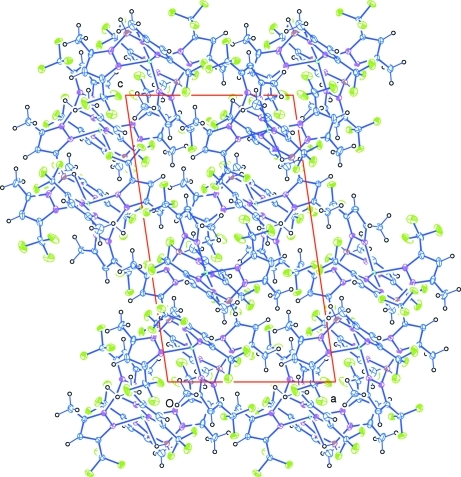
The crystal structure projected along the b cell direction. H atoms have been omitted for clarity.

## supplementary crystallographic information

### Comment

Our long-term interest in the synthesis and applications of nitrogen-containing heterocyles, such as expanded-ring imidazoles, and their complexes with metal ions (Stibrany & Potenza, 2009a) led us to prepare the title compound (I). With pyrazole, a variety of metal complexes has been prepared, predominantly with copper (Stibrany & Potenza, 2006), including the Cu(II) complex of an unusual (dimethylamino)methane bridged bis(pyrazole) ligand formed via a DMF aminalization (Stibrany *et al.*, 1999) and a novel binuclear µ-oxalato(1-benzylpyrazole)_2_(CF_3_SO_3_)_2_copper(II) compound resulting from the fixation of carbon dioxide (Stibrany *et al.*, 2005). A tris(pyrazolyl)arene ligand which forms geometrically constrained metal complexes has also been prepared (Stibrany *et al.*, 2006),as has a sterically strained trigonal-bipyramidal Cu(II) complex containing a 1-benzylpyrazole ligand (Stibrany & Potenza, 2009b). Copper and cobalt complexes utlizing the title ligand were prepared for oxidation studies (Gorun *et al.*, 2000).

Compound (I) (Fig. 1) contains a central Co^II^ ion linked to a Tp and a Bp ligand. Ligation is effected by three imine N atoms of the Tp ligand, two imine N atoms of the Bp ligand, and an H atom which participates in a two-electron, three-center B—H···Co bond. The result is a distorted octahedral coordination geometry.

A number of cobalt(II) complexes containing mixed bis (Bp)- and tris pyrazolylborates (Tp) and C—H···Co agostic interactions have been reported, with H···Co distances ranging from 2.03 Å (Ruman *et al.*, 2002), 2.035 Å (Ruman *et al.*, 2001) to 2.334 Å (Siemer *et al.*., 2001). The H···Co distance in (I), 2.17 Å, lies between these two extremes. Infrared spectral results also support the existence of an agostic 2-electron, 3-center bond in (I). B—H stretching vibrations for BH_2_ groups in which one of the H atoms is involved in a 2-electron, 3-center bond with a metal ion typically lie in the range 2100-2500 cm^-1^, with the lower value corresponding to the agostic interaction (Ghosh *et al.*, 1998). In (I), IR bands at 2569 and 2483 cm^-1^ are assigned as free B—H stretching vibrations, while the band at 2223 cm^-1 ^is assigned to the bound B—H group.

In the crystal, the molecules form layers parallel to the (10 -1) planes (Figures 2 and 3), which are linked by intermolecular C—H···F hydrogen bonds (Table 1) in such a way as to form chains along the a cell direction. Platon (Spek, 2009) reveals11 short intermolecular contacts, 10 of which are C—H···F interactions, which, along with the C—H···F hydrogen bonds noted above suggest that the trifluoromethyl groups in (I) contribute substantially to the stability of the structure.

### Experimental

Both Tp and Bp ligands were prepared as previously reported (Gorun *et al*., 2000). To a flask containing 10 ml of acetonitrile, 70 mg of Co(ClO_4_)_2_^.^6 H_2_O (0.19 mmol) was disssolved to give a pink solution. Then 94 mg of Tp (0.19 mmol) was added and the mixture was allowed to stir for 10 min. giving some white precipitate. Then, 67 mg of Bp (0.19 mmol) was added and the mixture was allowed to stir for an additional 15 min. The mixture was filtered to remove white solids and yielded a clear red solution, which was left to evaporate slowly. Upon evaporation, a major dichroic red-orange phase (compound (I)) was separated mechanically and characterized. IR (KBr pellet, cm^-1^); 2569(Tp, B—H, w), 2483(Bp, B—H, w), 2223(Bp, B—H···Co, w), 1471(s), 1261(s), 1165(s), 1114(s), 999(s), 795(m), 648(m).

### Refinement

Hydrogen atoms of the methyl groups were located on difference Fourier maps, those of the pyrrole fragments were positioned geometrically. For refinement a riding model was used, with C—H = 0.98 Å for methyl H atoms and 0.95 Å for pyrrole H atoms and *U*_iso_(H) = 1.2 *U*_eq _(C) and 1.5 *U*_eq _(C-methyl). Atom positions and isotropic displacement parameters of B—H hydrogens were refined freely.

### Crystal data

**Table:**

[Co(C_10_H_10_BF_6_N_4_)(C_15_H_13_BF_9_N_6_)]	*F*(000) = 1660
*M**_r_* = 829.08	*D*_x_ = 1.663 Mg m^−^^3^
Monoclinic, *P*2_1_/*n*	Mo *K*α radiation, λ = 0.71073 Å
Hall symbol: -P 2yn	Cell parameters from 952 reflections
*a* = 10.8195 (16) Å	θ = 2.6–26.7°
*b* = 16.559 (2) Å	µ = 0.64 mm^−^^1^
*c* = 18.687 (3) Å	*T* = 100 K
β = 98.408 (3)°	Block, orange-red
*V* = 3312.0 (8) Å^3^	0.37 × 0.30 × 0.14 mm
*Z* = 4	

### Data collection

**Table:**

Bruker SMART CCD area-detector diffractometer	7286 independent reflections
Radiation source: fine-focus sealed tube	6292 reflections with *I* > 2σ(*I*)
graphite	*R*_int_ = 0.024
φ and ω scans	θ_max_ = 27.1°, θ_min_ = 2.1°
Absorption correction: multi-scan (*SADABS*; Blessing, 1995)	*h* = −13→13
*T*_min_ = 0.919, *T*_max_ = 1.00	*k* = −21→20
33535 measured reflections	*l* = −23→23

### Refinement

**Table:**

Refinement on *F*^2^	Primary atom site location: structure-invariant direct methods
Least-squares matrix: full	Secondary atom site location: difference Fourier map
*R*[*F*^2^ > 2σ(*F*^2^)] = 0.039	Hydrogen site location: inferred from neighbouring sites
*wR*(*F*^2^) = 0.108	H atoms treated by a mixture of independent and constrained refinement
*S* = 1.00	*w* = 1/[σ^2^(*F*_o_^2^) + (0.0612*P*)^2^ + 2.660*P*] where *P* = (*F*_o_^2^ + 2*F*_c_^2^)/3
7286 reflections	(Δ/σ)_max_ = 0.002
495 parameters	Δρ_max_ = 0.69 e Å^−^^3^
0 restraints	Δρ_min_ = −0.44 e Å^−^^3^

### Special details

**Table:**

Geometry. All esds (except the esd in the dihedral angle between two l.s. planes) are estimated using the full covariance matrix. The cell esds are taken into account individually in the estimation of esds in distances, angles and torsion angles; correlations between esds in cell parameters are only used when they are defined by crystal symmetry. An approximate (isotropic) treatment of cell esds is used for estimating esds involving l.s. planes.
Refinement. Refinement of *F*^2^ against ALL reflections. The weighted *R*-factor wR and goodness of fit *S* are based on *F*^2^, conventional *R*-factors *R* are based on *F*, with *F* set to zero for negative *F*^2^. The threshold expression of *F*^2^ > σ(*F*^2^) is used only for calculating *R*-factors(gt) etc. and is not relevant to the choice of reflections for refinement. *R*-factors based on *F*^2^ are statistically about twice as large as those based on *F*, and *R*- factors based on ALL data will be even larger.

### Fractional atomic coordinates and isotropic or equivalent isotropic displacement parameters (Å^2^)

**Table:**

	*x*	*y*	*z*	*U*_iso_*/*U*_eq_	
Co1	0.67692 (2)	0.302118 (14)	0.620671 (13)	0.01918 (8)	
F1	1.04898 (14)	0.32294 (9)	0.81839 (7)	0.0442 (4)	
F2	0.97407 (14)	0.41514 (8)	0.74488 (7)	0.0416 (3)	
F3	0.85041 (13)	0.33420 (10)	0.78867 (8)	0.0435 (3)	
F4	0.81154 (15)	0.11513 (9)	0.37998 (8)	0.0467 (4)	
F5	0.97767 (14)	0.18564 (11)	0.40862 (8)	0.0523 (4)	
F6	0.85028 (15)	0.21414 (10)	0.31233 (7)	0.0482 (4)	
F7	0.54586 (13)	0.19079 (9)	0.75728 (8)	0.0438 (3)	
F8	0.44043 (15)	0.08179 (9)	0.73202 (10)	0.0535 (4)	
F9	0.38739 (12)	0.19101 (9)	0.67404 (9)	0.0455 (4)	
F10	0.99728 (15)	0.51996 (12)	0.60742 (11)	0.0658 (5)	
F11	0.86413 (16)	0.50824 (13)	0.51206 (10)	0.0670 (5)	
F12	0.93241 (13)	0.40309 (9)	0.56911 (9)	0.0451 (4)	
F13	0.44364 (19)	0.19865 (10)	0.50002 (10)	0.0662 (5)	
F14	0.25362 (17)	0.23531 (14)	0.47103 (13)	0.0826 (7)	
F15	0.4003 (2)	0.29983 (10)	0.43341 (10)	0.0603 (5)	
N1	0.86727 (15)	0.26325 (9)	0.65305 (8)	0.0205 (3)	
N2	0.90879 (15)	0.20741 (9)	0.60846 (9)	0.0220 (3)	
N3	0.70332 (16)	0.29844 (10)	0.51241 (9)	0.0234 (3)	
N4	0.77009 (16)	0.23567 (10)	0.49095 (9)	0.0248 (3)	
N5	0.64502 (15)	0.17499 (10)	0.62351 (9)	0.0241 (3)	
N6	0.70838 (16)	0.13117 (10)	0.57879 (9)	0.0253 (3)	
N7	0.72691 (15)	0.42384 (10)	0.64107 (9)	0.0228 (3)	
N8	0.65158 (16)	0.45466 (10)	0.68672 (9)	0.0265 (4)	
N9	0.48358 (16)	0.32725 (11)	0.60129 (10)	0.0277 (4)	
N10	0.45355 (16)	0.37470 (11)	0.65569 (10)	0.0306 (4)	
C1	0.95858 (19)	0.33758 (13)	0.76308 (11)	0.0268 (4)	
C2	0.96620 (18)	0.28046 (11)	0.70255 (10)	0.0218 (4)	
C3	1.07128 (18)	0.23733 (12)	0.69001 (11)	0.0256 (4)	
H3	1.1527	0.2394	0.7170	0.031*	
C4	1.03149 (19)	0.19085 (12)	0.62981 (11)	0.0266 (4)	
C5	1.1019 (2)	0.12976 (16)	0.59297 (14)	0.0402 (6)	
H5A	1.0657	0.0761	0.5978	0.060*	
H5B	1.1897	0.1296	0.6153	0.060*	
H5C	1.0965	0.1435	0.5416	0.060*	
C6	0.8573 (2)	0.19030 (15)	0.38120 (12)	0.0370 (5)	
C7	0.7869 (2)	0.24756 (14)	0.42153 (11)	0.0297 (4)	
C8	0.7303 (2)	0.31839 (14)	0.39755 (11)	0.0320 (5)	
H8	0.7273	0.3419	0.3510	0.038*	
C9	0.67811 (19)	0.34874 (12)	0.45617 (11)	0.0267 (4)	
C10	0.6041 (2)	0.42469 (13)	0.45943 (12)	0.0321 (5)	
H10A	0.5714	0.4271	0.5056	0.048*	
H10B	0.5344	0.4252	0.4195	0.048*	
H10C	0.6580	0.4715	0.4553	0.048*	
C11	0.4863 (2)	0.14675 (14)	0.70263 (13)	0.0349 (5)	
C12	0.56841 (19)	0.12256 (13)	0.64936 (12)	0.0294 (4)	
C13	0.5808 (2)	0.04605 (13)	0.62153 (14)	0.0385 (5)	
H13	0.5364	−0.0013	0.6311	0.046*	
C14	0.6711 (2)	0.05289 (13)	0.57699 (13)	0.0350 (5)	
C15	0.7250 (3)	−0.01038 (15)	0.53330 (17)	0.0528 (7)	
H15A	0.7172	0.0071	0.4827	0.079*	
H15B	0.6797	−0.0613	0.5362	0.079*	
H15C	0.8134	−0.0184	0.5524	0.079*	
C16	0.8966 (2)	0.47835 (14)	0.57809 (13)	0.0349 (5)	
C17	0.79552 (19)	0.48610 (12)	0.62379 (12)	0.0281 (4)	
C18	0.7643 (2)	0.55635 (13)	0.65694 (14)	0.0392 (6)	
H18	0.7989	0.6086	0.6530	0.047*	
C19	0.6729 (2)	0.53452 (14)	0.69650 (13)	0.0362 (5)	
C20	0.6035 (3)	0.58409 (19)	0.74419 (18)	0.0596 (9)	
H20A	0.5154	0.5876	0.7228	0.089*	
H20B	0.6395	0.6385	0.7491	0.089*	
H20C	0.6100	0.5587	0.7920	0.089*	
C21	0.3685 (2)	0.26228 (14)	0.49148 (15)	0.0430 (6)	
C22	0.3755 (2)	0.31429 (13)	0.55705 (14)	0.0347 (5)	
C23	0.2776 (2)	0.35359 (15)	0.58241 (15)	0.0414 (6)	
H23	0.1924	0.3542	0.5611	0.050*	
C24	0.3302 (2)	0.39173 (15)	0.64533 (14)	0.0392 (6)	
C25	0.2719 (3)	0.44475 (19)	0.69549 (16)	0.0544 (8)	
H25A	0.2817	0.4201	0.7437	0.082*	
H25B	0.1829	0.4514	0.6773	0.082*	
H25C	0.3129	0.4977	0.6985	0.082*	
B1	0.8187 (2)	0.16910 (14)	0.54601 (12)	0.0250 (4)	
B2	0.5641 (3)	0.39322 (17)	0.71633 (14)	0.0342 (5)	
H1B	0.865 (2)	0.1208 (14)	0.5190 (12)	0.021 (5)*	
H21B	0.537 (2)	0.4097 (16)	0.7632 (14)	0.039 (7)*	
H22B	0.623 (2)	0.3326 (16)	0.7245 (13)	0.034 (6)*	

### Atomic displacement parameters (Å^2^)

**Table:**

	*U*^11^	*U*^22^	*U*^33^	*U*^12^	*U*^13^	*U*^23^
Co1	0.01869 (13)	0.01639 (13)	0.02269 (14)	0.00083 (9)	0.00378 (9)	0.00099 (9)
F1	0.0457 (8)	0.0479 (8)	0.0336 (7)	0.0131 (7)	−0.0119 (6)	−0.0087 (6)
F2	0.0560 (9)	0.0245 (6)	0.0418 (8)	−0.0014 (6)	−0.0008 (6)	−0.0054 (5)
F3	0.0374 (7)	0.0564 (9)	0.0388 (7)	−0.0033 (7)	0.0125 (6)	−0.0156 (7)
F4	0.0610 (10)	0.0411 (8)	0.0383 (8)	0.0064 (7)	0.0084 (7)	−0.0108 (6)
F5	0.0371 (8)	0.0768 (12)	0.0437 (8)	0.0101 (7)	0.0081 (6)	−0.0089 (8)
F6	0.0538 (9)	0.0634 (10)	0.0296 (7)	0.0059 (8)	0.0139 (6)	−0.0045 (6)
F7	0.0353 (7)	0.0552 (9)	0.0426 (8)	0.0054 (6)	0.0120 (6)	0.0043 (7)
F8	0.0480 (9)	0.0410 (8)	0.0773 (11)	0.0004 (7)	0.0284 (8)	0.0288 (8)
F9	0.0249 (7)	0.0527 (9)	0.0593 (9)	0.0102 (6)	0.0081 (6)	0.0264 (7)
F10	0.0372 (8)	0.0734 (12)	0.0866 (13)	−0.0277 (8)	0.0084 (8)	−0.0085 (10)
F11	0.0490 (9)	0.0924 (14)	0.0610 (10)	0.0132 (9)	0.0128 (8)	0.0483 (10)
F12	0.0412 (8)	0.0387 (8)	0.0615 (9)	0.0026 (6)	0.0275 (7)	0.0110 (7)
F13	0.0834 (13)	0.0372 (8)	0.0654 (11)	0.0137 (8)	−0.0310 (10)	−0.0072 (7)
F14	0.0451 (10)	0.0953 (16)	0.1006 (16)	−0.0343 (10)	−0.0126 (10)	−0.0172 (13)
F15	0.0841 (13)	0.0401 (9)	0.0539 (10)	−0.0147 (8)	0.0003 (9)	0.0004 (7)
N1	0.0219 (8)	0.0167 (7)	0.0234 (8)	0.0002 (6)	0.0045 (6)	0.0006 (6)
N2	0.0222 (8)	0.0202 (8)	0.0242 (8)	0.0011 (6)	0.0054 (6)	−0.0018 (6)
N3	0.0248 (8)	0.0203 (8)	0.0248 (8)	−0.0004 (6)	0.0021 (6)	−0.0006 (6)
N4	0.0255 (8)	0.0244 (8)	0.0243 (8)	−0.0003 (7)	0.0032 (6)	−0.0037 (6)
N5	0.0205 (8)	0.0211 (8)	0.0304 (8)	−0.0010 (6)	0.0027 (6)	0.0038 (6)
N6	0.0271 (8)	0.0178 (8)	0.0304 (8)	−0.0009 (6)	0.0019 (7)	−0.0011 (6)
N7	0.0228 (8)	0.0212 (8)	0.0244 (8)	0.0026 (6)	0.0038 (6)	−0.0006 (6)
N8	0.0271 (8)	0.0262 (8)	0.0253 (8)	0.0080 (7)	0.0011 (7)	−0.0070 (7)
N9	0.0216 (8)	0.0248 (8)	0.0369 (9)	−0.0010 (7)	0.0047 (7)	0.0060 (7)
N10	0.0249 (9)	0.0298 (9)	0.0402 (10)	0.0044 (7)	0.0147 (7)	0.0106 (8)
C1	0.0252 (10)	0.0261 (10)	0.0279 (10)	0.0021 (8)	−0.0003 (8)	−0.0008 (8)
C2	0.0224 (9)	0.0191 (8)	0.0238 (9)	−0.0027 (7)	0.0031 (7)	0.0037 (7)
C3	0.0198 (9)	0.0264 (10)	0.0307 (10)	−0.0013 (7)	0.0037 (7)	0.0025 (8)
C4	0.0221 (9)	0.0284 (10)	0.0302 (10)	0.0025 (8)	0.0069 (8)	0.0017 (8)
C5	0.0276 (11)	0.0487 (14)	0.0445 (13)	0.0109 (10)	0.0055 (10)	−0.0130 (11)
C6	0.0374 (12)	0.0458 (14)	0.0283 (11)	0.0024 (10)	0.0067 (9)	−0.0042 (9)
C7	0.0298 (10)	0.0347 (11)	0.0242 (9)	−0.0041 (8)	0.0028 (8)	−0.0039 (8)
C8	0.0362 (12)	0.0354 (12)	0.0240 (10)	−0.0036 (9)	0.0031 (8)	0.0014 (8)
C9	0.0279 (10)	0.0262 (10)	0.0245 (9)	−0.0050 (8)	−0.0013 (8)	0.0011 (8)
C10	0.0399 (12)	0.0243 (10)	0.0305 (10)	0.0009 (9)	−0.0002 (9)	0.0055 (8)
C11	0.0245 (10)	0.0343 (12)	0.0464 (13)	0.0023 (9)	0.0069 (9)	0.0194 (10)
C12	0.0230 (10)	0.0262 (10)	0.0376 (11)	−0.0021 (8)	−0.0001 (8)	0.0115 (8)
C13	0.0376 (12)	0.0232 (10)	0.0530 (14)	−0.0079 (9)	0.0008 (11)	0.0074 (10)
C14	0.0378 (12)	0.0211 (10)	0.0443 (12)	−0.0057 (9)	0.0002 (10)	0.0005 (9)
C15	0.0657 (18)	0.0217 (11)	0.0725 (19)	−0.0089 (11)	0.0149 (15)	−0.0126 (12)
C16	0.0261 (10)	0.0325 (11)	0.0448 (13)	−0.0050 (9)	0.0008 (9)	0.0137 (10)
C17	0.0236 (9)	0.0210 (9)	0.0368 (11)	−0.0007 (7)	−0.0051 (8)	0.0030 (8)
C18	0.0309 (11)	0.0203 (10)	0.0602 (15)	−0.0005 (8)	−0.0143 (10)	−0.0047 (10)
C19	0.0305 (11)	0.0283 (11)	0.0445 (13)	0.0090 (9)	−0.0122 (9)	−0.0152 (9)
C20	0.0492 (16)	0.0520 (17)	0.0720 (19)	0.0197 (13)	−0.0102 (14)	−0.0396 (15)
C21	0.0359 (12)	0.0273 (11)	0.0590 (15)	−0.0096 (9)	−0.0161 (11)	0.0102 (11)
C22	0.0231 (10)	0.0267 (11)	0.0525 (14)	−0.0043 (8)	−0.0007 (9)	0.0164 (9)
C23	0.0195 (10)	0.0385 (13)	0.0656 (16)	0.0001 (9)	0.0042 (10)	0.0263 (12)
C24	0.0259 (11)	0.0386 (12)	0.0566 (15)	0.0087 (9)	0.0182 (10)	0.0266 (11)
C25	0.0419 (14)	0.0625 (18)	0.0663 (18)	0.0238 (13)	0.0327 (13)	0.0268 (15)
B1	0.0253 (11)	0.0230 (10)	0.0264 (11)	0.0009 (8)	0.0028 (9)	−0.0028 (8)
B2	0.0352 (13)	0.0410 (14)	0.0286 (12)	0.0114 (11)	0.0118 (10)	0.0064 (10)

### Geometric parameters (Å, °)

**Table:**

Co1—N3	2.0850 (17)	C2—C3	1.391 (3)
Co1—N7	2.1072 (17)	C3—C4	1.379 (3)
Co1—N9	2.1115 (18)	C3—H3	0.9500
Co1—N5	2.1351 (17)	C4—C5	1.493 (3)
Co1—N1	2.1581 (16)	C5—H5A	0.9800
Co1—H22B	2.17 (2)	C5—H5B	0.9800
F1—C1	1.337 (2)	C5—H5C	0.9800
F2—C1	1.345 (2)	C6—C7	1.489 (3)
F3—C1	1.329 (2)	C7—C8	1.368 (3)
F4—C6	1.339 (3)	C8—C9	1.397 (3)
F5—C6	1.330 (3)	C8—H8	0.9500
F6—C6	1.338 (3)	C9—C10	1.497 (3)
F7—C11	1.341 (3)	C10—H10A	0.9800
F8—C11	1.336 (2)	C10—H10B	0.9800
F9—C11	1.342 (2)	C10—H10C	0.9800
F10—C16	1.337 (3)	C11—C12	1.483 (3)
F11—C16	1.328 (3)	C12—C13	1.384 (3)
F12—C16	1.323 (3)	C13—C14	1.377 (3)
F13—C21	1.326 (3)	C13—H13	0.9500
F14—C21	1.323 (3)	C14—C15	1.498 (3)
F15—C21	1.339 (3)	C15—H15A	0.9800
N1—C2	1.339 (2)	C15—H15B	0.9800
N1—N2	1.364 (2)	C15—H15C	0.9800
N2—C4	1.357 (3)	C16—C17	1.488 (3)
N2—B1	1.543 (3)	C17—C18	1.383 (3)
N3—C9	1.337 (3)	C18—C19	1.367 (4)
N3—N4	1.359 (2)	C18—H18	0.9500
N4—C7	1.350 (3)	C19—C20	1.493 (3)
N4—B1	1.547 (3)	C20—H20A	0.9800
N5—C12	1.338 (3)	C20—H20B	0.9800
N5—N6	1.365 (2)	C20—H20C	0.9800
N6—C14	1.357 (3)	C21—C22	1.490 (4)
N6—B1	1.551 (3)	C22—C23	1.384 (3)
N7—C17	1.337 (3)	C23—C24	1.382 (4)
N7—N8	1.362 (2)	C23—H23	0.9500
N8—C19	1.350 (3)	C24—C25	1.490 (4)
N8—B2	1.546 (3)	C25—H25A	0.9800
N9—C22	1.347 (3)	C25—H25B	0.9800
N9—N10	1.361 (3)	C25—H25C	0.9800
N10—C24	1.350 (3)	B1—H1B	1.10 (2)
N10—B2	1.554 (3)	B2—H21B	1.00 (3)
C1—C2	1.486 (3)	B2—H22B	1.18 (3)
			
N3—Co1—N7	97.79 (6)	C9—C10—H10B	109.5
N3—Co1—N9	96.44 (7)	H10A—C10—H10B	109.5
N7—Co1—N9	93.68 (7)	C9—C10—H10C	109.5
N3—Co1—N5	92.34 (6)	H10A—C10—H10C	109.5
N7—Co1—N5	167.62 (6)	H10B—C10—H10C	109.5
N9—Co1—N5	92.23 (7)	F8—C11—F7	106.91 (19)
N3—Co1—N1	90.16 (6)	F8—C11—F9	106.34 (17)
N7—Co1—N1	91.45 (6)	F7—C11—F9	105.8 (2)
N9—Co1—N1	171.01 (6)	F8—C11—C12	110.7 (2)
N5—Co1—N1	81.38 (6)	F7—C11—C12	112.91 (18)
N3—Co1—H22B	165.7 (7)	F9—C11—C12	113.67 (19)
N7—Co1—H22B	73.2 (7)	N5—C12—C13	111.1 (2)
N9—Co1—H22B	73.5 (7)	N5—C12—C11	122.0 (2)
N5—Co1—H22B	98.1 (7)	C13—C12—C11	126.9 (2)
N1—Co1—H22B	101.0 (7)	C14—C13—C12	105.54 (19)
C2—N1—N2	105.35 (15)	C14—C13—H13	127.2
C2—N1—Co1	139.81 (13)	C12—C13—H13	127.2
N2—N1—Co1	114.64 (12)	N6—C14—C13	107.4 (2)
C4—N2—N1	110.66 (16)	N6—C14—C15	122.9 (2)
C4—N2—B1	128.46 (16)	C13—C14—C15	129.7 (2)
N1—N2—B1	120.82 (15)	C14—C15—H15A	109.5
C9—N3—N4	107.49 (16)	C14—C15—H15B	109.5
C9—N3—Co1	134.68 (14)	H15A—C15—H15B	109.5
N4—N3—Co1	117.56 (12)	C14—C15—H15C	109.5
C7—N4—N3	108.89 (17)	H15A—C15—H15C	109.5
C7—N4—B1	131.52 (17)	H15B—C15—H15C	109.5
N3—N4—B1	119.49 (16)	F12—C16—F11	106.0 (2)
C12—N5—N6	105.62 (16)	F12—C16—F10	107.54 (19)
C12—N5—Co1	139.42 (15)	F11—C16—F10	106.13 (19)
N6—N5—Co1	114.30 (12)	F12—C16—C17	114.03 (17)
C14—N6—N5	110.41 (17)	F11—C16—C17	112.59 (19)
C14—N6—B1	128.35 (18)	F10—C16—C17	110.1 (2)
N5—N6—B1	120.71 (16)	N7—C17—C18	111.1 (2)
C17—N7—N8	105.45 (16)	N7—C17—C16	123.54 (19)
C17—N7—Co1	146.16 (14)	C18—C17—C16	125.3 (2)
N8—N7—Co1	108.08 (12)	C19—C18—C17	105.3 (2)
C19—N8—N7	110.21 (18)	C19—C18—H18	127.4
C19—N8—B2	134.25 (19)	C17—C18—H18	127.4
N7—N8—B2	115.54 (16)	N8—C19—C18	107.99 (19)
C22—N9—N10	105.65 (18)	N8—C19—C20	121.9 (3)
C22—N9—Co1	145.42 (17)	C18—C19—C20	130.1 (2)
N10—N9—Co1	108.92 (13)	C19—C20—H20A	109.5
C24—N10—N9	110.6 (2)	C19—C20—H20B	109.5
C24—N10—B2	134.7 (2)	H20A—C20—H20B	109.5
N9—N10—B2	114.60 (16)	C19—C20—H20C	109.5
F3—C1—F1	107.30 (17)	H20A—C20—H20C	109.5
F3—C1—F2	106.26 (17)	H20B—C20—H20C	109.5
F1—C1—F2	105.33 (17)	F14—C21—F13	107.5 (2)
F3—C1—C2	113.39 (17)	F14—C21—F15	105.4 (2)
F1—C1—C2	110.93 (17)	F13—C21—F15	103.9 (2)
F2—C1—C2	113.11 (17)	F14—C21—C22	111.4 (2)
N1—C2—C3	111.40 (17)	F13—C21—C22	113.8 (2)
N1—C2—C1	122.25 (17)	F15—C21—C22	114.20 (19)
C3—C2—C1	126.34 (18)	N9—C22—C23	110.7 (2)
C4—C3—C2	104.99 (18)	N9—C22—C21	122.1 (2)
C4—C3—H3	127.5	C23—C22—C21	127.1 (2)
C2—C3—H3	127.5	C24—C23—C22	105.4 (2)
N2—C4—C3	107.60 (17)	C24—C23—H23	127.3
N2—C4—C5	122.97 (19)	C22—C23—H23	127.3
C3—C4—C5	129.40 (19)	N10—C24—C23	107.6 (2)
C4—C5—H5A	109.5	N10—C24—C25	122.2 (3)
C4—C5—H5B	109.5	C23—C24—C25	130.2 (2)
H5A—C5—H5B	109.5	C24—C25—H25A	109.5
C4—C5—H5C	109.5	C24—C25—H25B	109.5
H5A—C5—H5C	109.5	H25A—C25—H25B	109.5
H5B—C5—H5C	109.5	C24—C25—H25C	109.5
F5—C6—F6	107.6 (2)	H25A—C25—H25C	109.5
F5—C6—F4	106.9 (2)	H25B—C25—H25C	109.5
F6—C6—F4	106.81 (19)	N2—B1—N4	109.02 (16)
F5—C6—C7	112.75 (19)	N2—B1—N6	107.77 (16)
F6—C6—C7	109.7 (2)	N4—B1—N6	110.19 (16)
F4—C6—C7	112.67 (19)	N2—B1—H1B	111.4 (12)
N4—C7—C8	108.87 (19)	N4—B1—H1B	110.1 (11)
N4—C7—C6	123.0 (2)	N6—B1—H1B	108.3 (12)
C8—C7—C6	128.2 (2)	N8—B2—N10	108.37 (17)
C7—C8—C9	105.33 (19)	N8—B2—H21B	113.9 (16)
C7—C8—H8	127.3	N10—B2—H21B	113.7 (15)
C9—C8—H8	127.3	N8—B2—H22B	105.1 (12)
N3—C9—C8	109.43 (19)	N10—B2—H22B	105.5 (12)
N3—C9—C10	122.69 (18)	H21B—B2—H22B	109.6 (19)
C8—C9—C10	127.88 (19)	B2—H22B—Co1	107 (1)
C9—C10—H10A	109.5		
			
N3—Co1—N1—C2	136.1 (2)	N3—N4—C7—C6	179.51 (19)
N7—Co1—N1—C2	38.3 (2)	B1—N4—C7—C6	3.4 (3)
N5—Co1—N1—C2	−131.5 (2)	F5—C6—C7—N4	−65.0 (3)
H22B—Co1—N1—C2	−34.9 (7)	F6—C6—C7—N4	175.1 (2)
N3—Co1—N1—N2	−37.84 (12)	F4—C6—C7—N4	56.2 (3)
N7—Co1—N1—N2	−135.64 (12)	F5—C6—C7—C8	114.7 (3)
N5—Co1—N1—N2	54.51 (12)	F6—C6—C7—C8	−5.3 (3)
H22B—Co1—N1—N2	151.2 (7)	F4—C6—C7—C8	−124.1 (2)
C2—N1—N2—C4	−0.4 (2)	N4—C7—C8—C9	−0.1 (2)
Co1—N1—N2—C4	175.57 (12)	C6—C7—C8—C9	−179.8 (2)
C2—N1—N2—B1	176.91 (16)	N4—N3—C9—C8	−0.5 (2)
Co1—N1—N2—B1	−7.1 (2)	Co1—N3—C9—C8	173.09 (15)
N7—Co1—N3—C9	−41.0 (2)	N4—N3—C9—C10	179.22 (18)
N9—Co1—N3—C9	53.6 (2)	Co1—N3—C9—C10	−7.2 (3)
N5—Co1—N3—C9	146.09 (19)	C7—C8—C9—N3	0.4 (2)
N1—Co1—N3—C9	−132.53 (19)	C7—C8—C9—C10	−179.3 (2)
H22B—Co1—N3—C9	9(3)	N6—N5—C12—C13	−0.5 (2)
N7—Co1—N3—N4	132.08 (13)	Co1—N5—C12—C13	168.92 (17)
N9—Co1—N3—N4	−133.29 (13)	N6—N5—C12—C11	177.70 (18)
N5—Co1—N3—N4	−40.79 (14)	Co1—N5—C12—C11	−12.9 (3)
N1—Co1—N3—N4	40.59 (14)	F8—C11—C12—N5	−166.19 (19)
H22B—Co1—N3—N4	−178 (3)	F7—C11—C12—N5	−46.4 (3)
C9—N3—N4—C7	0.4 (2)	F9—C11—C12—N5	74.2 (3)
Co1—N3—N4—C7	−174.44 (13)	F8—C11—C12—C13	11.7 (3)
C9—N3—N4—B1	177.10 (17)	F7—C11—C12—C13	131.5 (2)
Co1—N3—N4—B1	2.2 (2)	F9—C11—C12—C13	−107.9 (3)
N3—Co1—N5—C12	−137.6 (2)	N5—C12—C13—C14	0.7 (3)
N7—Co1—N5—C12	77.4 (4)	C11—C12—C13—C14	−177.4 (2)
N9—Co1—N5—C12	−41.1 (2)	N5—N6—C14—C13	0.2 (2)
N1—Co1—N5—C12	132.6 (2)	B1—N6—C14—C13	171.8 (2)
H22B—Co1—N5—C12	32.6 (7)	N5—N6—C14—C15	−179.2 (2)
N3—Co1—N5—N6	31.20 (13)	B1—N6—C14—C15	−7.6 (4)
N7—Co1—N5—N6	−113.8 (3)	C12—C13—C14—N6	−0.5 (3)
N9—Co1—N5—N6	127.74 (13)	C12—C13—C14—C15	178.9 (3)
N1—Co1—N5—N6	−58.61 (13)	N8—N7—C17—C18	−0.7 (2)
H22B—Co1—N5—N6	−158.6 (7)	Co1—N7—C17—C18	171.47 (18)
C12—N5—N6—C14	0.2 (2)	N8—N7—C17—C16	176.13 (18)
Co1—N5—N6—C14	−172.32 (14)	Co1—N7—C17—C16	−11.7 (4)
C12—N5—N6—B1	−172.15 (17)	F12—C16—C17—N7	−14.3 (3)
Co1—N5—N6—B1	15.4 (2)	F11—C16—C17—N7	106.5 (2)
N3—Co1—N7—C17	−28.7 (2)	F10—C16—C17—N7	−135.3 (2)
N9—Co1—N7—C17	−125.7 (2)	F12—C16—C17—C18	162.0 (2)
N5—Co1—N7—C17	116.0 (3)	F11—C16—C17—C18	−77.1 (3)
N1—Co1—N7—C17	61.7 (2)	F10—C16—C17—C18	41.1 (3)
H22B—Co1—N7—C17	162.7 (7)	N7—C17—C18—C19	0.5 (3)
N3—Co1—N7—N8	143.38 (12)	C16—C17—C18—C19	−176.3 (2)
N9—Co1—N7—N8	46.36 (12)	N7—N8—C19—C18	−0.3 (2)
N5—Co1—N7—N8	−72.0 (3)	B2—N8—C19—C18	178.6 (2)
N1—Co1—N7—N8	−126.26 (12)	N7—N8—C19—C20	−179.9 (2)
H22B—Co1—N7—N8	−25.2 (7)	B2—N8—C19—C20	−0.9 (4)
C17—N7—N8—C19	0.6 (2)	C17—C18—C19—N8	−0.1 (2)
Co1—N7—N8—C19	−174.78 (13)	C17—C18—C19—C20	179.4 (2)
C17—N7—N8—B2	−178.54 (17)	N10—N9—C22—C23	0.6 (2)
Co1—N7—N8—B2	6.0 (2)	Co1—N9—C22—C23	178.91 (19)
N3—Co1—N9—C22	34.9 (3)	N10—N9—C22—C21	−178.06 (19)
N7—Co1—N9—C22	133.2 (3)	Co1—N9—C22—C21	0.3 (4)
N5—Co1—N9—C22	−57.7 (3)	F14—C21—C22—N9	158.1 (2)
H22B—Co1—N9—C22	−155.5 (8)	F13—C21—C22—N9	36.4 (3)
N3—Co1—N9—N10	−146.81 (12)	F15—C21—C22—N9	−82.7 (3)
N7—Co1—N9—N10	−48.53 (13)	F14—C21—C22—C23	−20.3 (3)
N5—Co1—N9—N10	120.58 (13)	F13—C21—C22—C23	−142.0 (2)
H22B—Co1—N9—N10	22.8 (7)	F15—C21—C22—C23	98.9 (3)
C22—N9—N10—C24	−0.7 (2)	N9—C22—C23—C24	−0.3 (2)
Co1—N9—N10—C24	−179.68 (13)	C21—C22—C23—C24	178.3 (2)
C22—N9—N10—B2	176.42 (17)	N9—N10—C24—C23	0.5 (2)
Co1—N9—N10—B2	−2.6 (2)	B2—N10—C24—C23	−175.8 (2)
N2—N1—C2—C3	0.8 (2)	N9—N10—C24—C25	−178.1 (2)
Co1—N1—C2—C3	−173.55 (14)	B2—N10—C24—C25	5.6 (4)
N2—N1—C2—C1	−177.99 (17)	C22—C23—C24—N10	−0.2 (2)
Co1—N1—C2—C1	7.7 (3)	C22—C23—C24—C25	178.3 (2)
F3—C1—C2—N1	35.8 (3)	C4—N2—B1—N4	−119.1 (2)
F1—C1—C2—N1	156.65 (18)	N1—N2—B1—N4	64.2 (2)
F2—C1—C2—N1	−85.3 (2)	C4—N2—B1—N6	121.4 (2)
F3—C1—C2—C3	−142.7 (2)	N1—N2—B1—N6	−55.4 (2)
F1—C1—C2—C3	−21.9 (3)	C7—N4—B1—N2	114.3 (2)
F2—C1—C2—C3	96.2 (2)	N3—N4—B1—N2	−61.4 (2)
N1—C2—C3—C4	−0.8 (2)	C7—N4—B1—N6	−127.6 (2)
C1—C2—C3—C4	177.86 (19)	N3—N4—B1—N6	56.6 (2)
N1—N2—C4—C3	−0.1 (2)	C14—N6—B1—N2	−120.1 (2)
B1—N2—C4—C3	−177.15 (18)	N5—N6—B1—N2	50.7 (2)
N1—N2—C4—C5	177.78 (19)	C14—N6—B1—N4	121.0 (2)
B1—N2—C4—C5	0.7 (3)	N5—N6—B1—N4	−68.2 (2)
C2—C3—C4—N2	0.6 (2)	C19—N8—B2—N10	104.5 (3)
C2—C3—C4—C5	−177.2 (2)	N7—N8—B2—N10	−76.6 (2)
N3—N4—C7—C8	−0.2 (2)	C24—N10—B2—N8	−110.2 (2)
B1—N4—C7—C8	−176.3 (2)	N9—N10—B2—N8	73.6 (2)

### Hydrogen-bond geometry (Å, °)

**Table:**

*D*—H···*A*	*D*—H	H···*A*	*D*···*A*	*D*—H···*A*
C5—H5B···F9^i^	0.98	2.48	3.391 (3)	155
C10—H10C···F11	0.98	2.40	3.160 (3)	134
C25—H25B···F10^ii^	0.98	2.50	3.414 (4)	155

Symmetry codes: (i) *x*+1, *y*, *z*; (ii) *x*−1, *y*, *z*.
